# The value of cardiac CT based inflammatory risk assessment in predicting cardiovascular events: a case report

**DOI:** 10.1186/s12872-025-05171-z

**Published:** 2025-10-08

**Authors:** Michail C. Mavrogiannis, Nerea Sanfeliu Garces, Lucas Costa, Ahmed Alsinbili, Attila Kardos

**Affiliations:** 1 Cardiovascular Medicine Division, Radcliffe Department of Medicine, University of Oxford, Oxford, UK; 2 Department of Cardiology, Translational Cardiovascular Research Group, Milton Keynes University Hospital NHS Foundation Trust, Milton Keynes, UK; 3 Faculty of Medicine and Health Sciences, University of Buckingham, Buckingham, UK

**Keywords:** Inflammation, Coronary artery disease, Computed tomography, Artificial intelligence

## Abstract

**Background:**

Vascular inflammation plays a critical role in the development of coronary artery disease (CAD). Measurement of coronary inflammation from coronary computed tomography angiography (CCTA) using the perivascular fat attenuation index (FAI) Score could provide unique prognostic information and guide the clinical management of patients. In this context, we also refer to an artificial intelligence–based risk prediction tool (AI-Risk algorithm), which integrates FAI Score with clinical risk factors and plaque burden to estimate the long-term probability of a fatal cardiac event.

**Case presentation:**

A 69-year-old male presented with symptoms of new onset angina. Past medical history included coronary artery bypass grafting (CABG) in 2001. Initial evaluation with CCTA showed patent arterial graft to left anterior descending (LAD) artery and two occluded venous grafts to obtuse marginal and diagonal branches, respectively, were identified. The non-grafted right coronary artery (RCA) was non-obstructive with moderate mid-vessel stenosis and the patient was discharged on optimal medical therapy. However, the patient was intolerant to statin. Eight years later, the patient was admitted to the hospital with a non-ST segment elevation myocardial infarction (NSTEMI) and the invasive coronary angiography showed occlusion of the non-grafted RCA. After few months of guidelines directed medical therapy, the patient developed progressive heart failure due to ischaemic cardiomyopathy and mitral regurgitation that led to his death. Retrospective perivascular FAI measurement of the non-grafted RCA captured the significantly elevated residual inflammatory risk.

**Conclusions:**

The utilization of perivascular FAI Score and AI-Risk algorithm to capture inflammatory risk and predict future events beyond the current clinical risk stratification and CCTA interpretation, especially in the absence of obstructive CAD, could offer an important adjunct to current strategies in preventive cardiology, pending further validation. In this case report, our patient’s management plan could have been adjusted had these technologies been available during initial evaluation, and the high inflammatory burden of the non-grafted RCA was timely captured.

## Introduction

Vascular inflammation plays a crucial role in the development and progression of atherosclerotic coronary artery disease (CAD). The perivascular fat attenuation index (FAI) uses coronary computed tomography angiography (CCTA) to quantify attenuation gradients in perivascular adipose tissue (PVAT) induced by inflamed coronary vessels [1]. FAI Score is a regulatory cleared metric of coronary inflammation which adjusts FAI values for age, sex, scan technical parameters, biological, and anatomical parameters, providing a standardized tool to individualize the inflammatory burden of each coronary artery [2]. FAI score measurements and patient risk factors were incorporated into a prognostic AI-Risk algorithm to generate the individualized 8-year % patient risk for a fatal cardiac event [2].

The AI-Risk algorithm was developed and validated using large-scale, multi-centre datasets, with model calibration incorporating demographic factors (age, sex), biological markers (e.g., lipid profiles), anatomical features (coronary territory and plaque characteristics), and scan acquisition parameters. Goodness-of-fit, calibration, and discrimination metrics for the model, along with techniques to prevent overfitting (e.g., cross-validation and independent test sets), have been detailed in previous studies [1, 2]. These principles were applied when the algorithm was used retrospectively in our case.

For the first time, we report a case where the retrospective measurement of perivascular FAI score and AI-Risk algorithm analysis of a non-grafted coronary artery shows that the increased risk for a future event could have been accurately predicted. The right coronary artery (RCA) was selected for retrospective analysis because it was the only major epicardial vessel not grafted during the patient’s CABG in 2001 and subsequently became the site of infarction in 2022, despite showing only moderate stenosis in the 2014 CCTA. The AI-Risk algorithm calculates the risk for a fatal cardiac event if no treatment is given, based on the FAI-Score values, the coronary atherosclerotic plaque burden and clinical risk factors which include age, sex, smoking status, hypercholesterolaemia, diabetes, and hypertension. This case report aims to highlight the clinical utility and predictive value of measuring coronary inflammation using perivascular FAI Score and the AI-Risk algorithm, even in the absence of obstructive CAD.

## Case presentation

### Initial evaluation

**Fig. 1:**
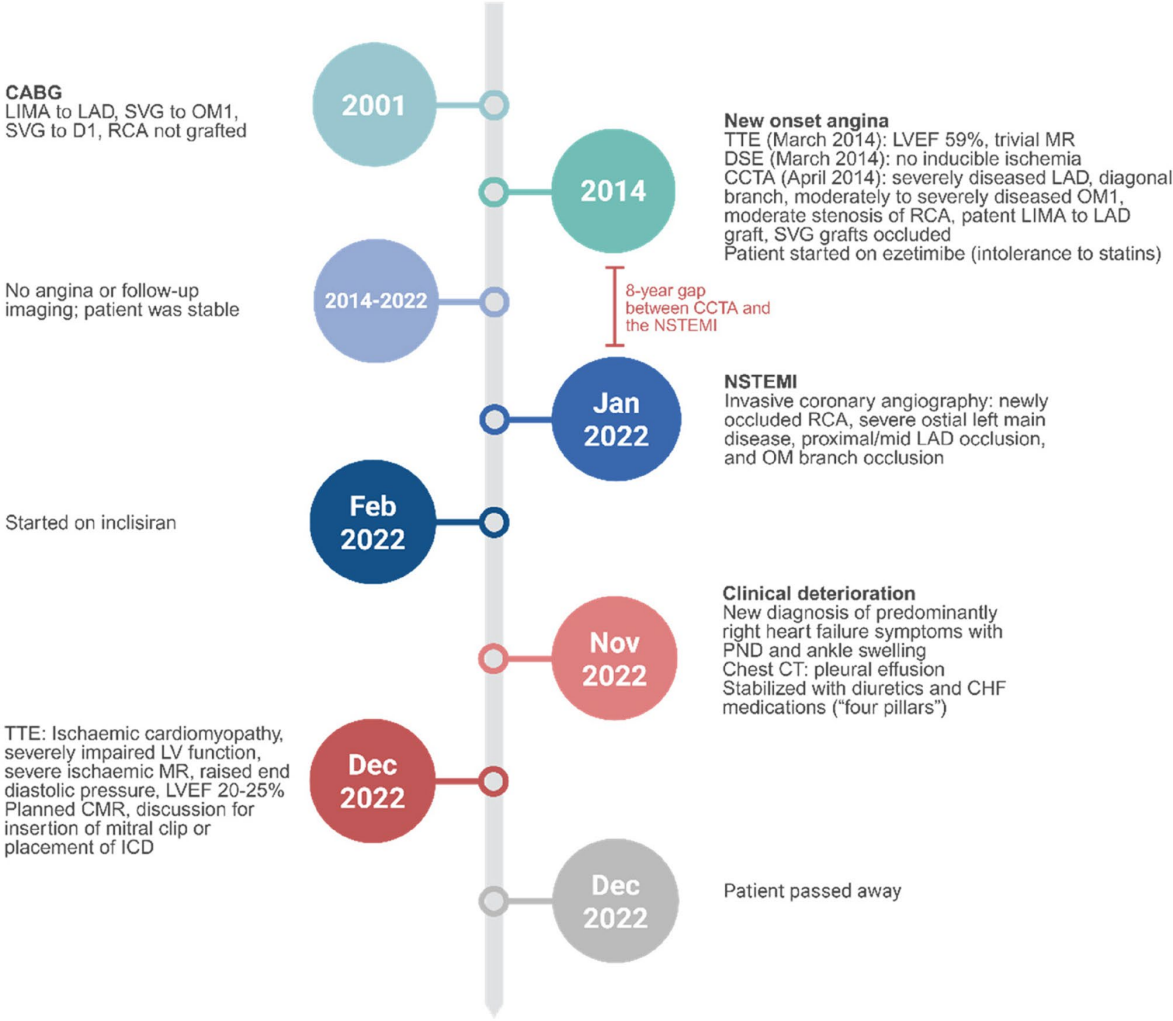
Schematic timeline summarizing key clinical events, imaging findings, and management decisions in the patient’s course from CABG in 2001, CCTA in April 2014, through the NSTEMI and development of heart failure in 2022

In 2014, a 69-year-old male patient sought medical attention due to new onset angina (Fig. 1). His past medical history included coronary artery bypass grafting (CABG) operation in 2001. He received a left internal mammary artery (LIMA) graft to the left anterior descending (LAD), and saphenous vein grafts (SVG) to the first obtuse marginal artery (OM1) and first diagonal branch (D1). The right coronary artery (RCA) was not grafted. His cardiovascular risk factors included hypercholesterolaemia, hypertension, and a positive family history. His medications at the time included clopidogrel 75 mg, felodipine 2.5 mg, pravastatin 40 mg, omeprazole 10 mg, and furosemide 20 mg.

**Fig. 2:**
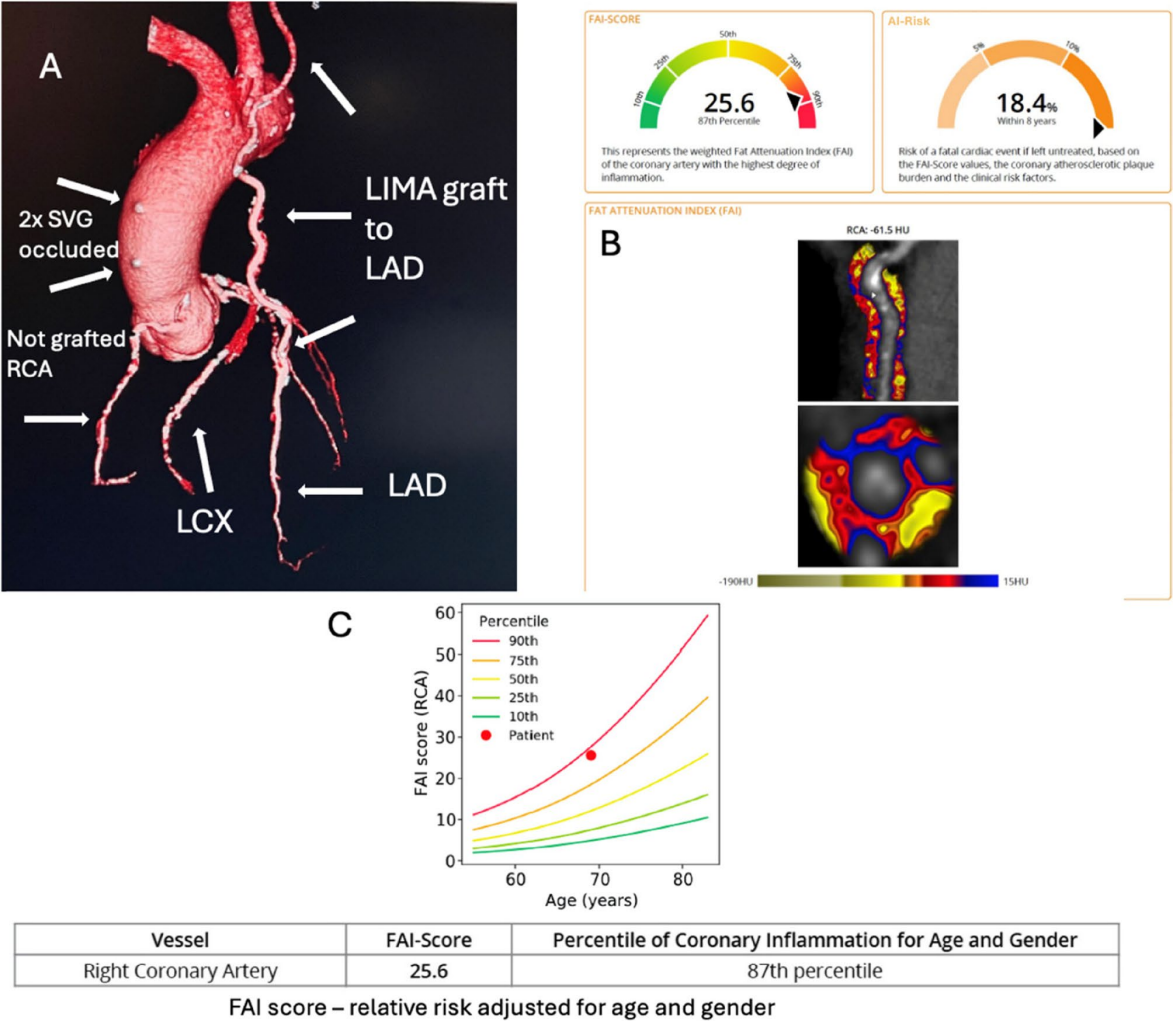
Coronary CT angiography and the FAI metrics

In order to identify any structural or functional heart abnormalities behind the anginal symptoms on a background of a previous triple CABG, a transthoracic echocardiogram (TTE) along with a dobutamine stress echocardiography (DSE) were performed. The TTE was within normal limits (ejection fraction 59%) with trivial mitral regurgitation (MR) while the DSE at high workload showed no inducible ischemia and then, the patient was scheduled for a CCTA. The CCTA showed a severely diseased LAD and diagonal branch, and moderately to severely stenosed OM1. The non-grafted RCA exhibited proximal external remodelling with some mixed plaque at the distal part of the proximal segment which was causing moderate stenosis (40–50%). Although not considered hemodynamically significant at the time, the RCA was the only major coronary artery that was not revascularized during prior CABG and was therefore clinically relevant for follow-up. This informed our decision to focus retrospective FAI analysis on the RCA. The LIMA to LAD graft was patent, while both of the SVG grafts have been occluded (Fig. 2A). Optimal medical therapy was recommended. An invasive coronary angiography was not indicated. Due to persistent statin intolerance with muscle aches and memory impairment, the patient was started on ezetimibe.

A 3D reconstruction of the 2014 CCTA scan showing severely diseased left anterior descending (LAD) and left circumflex (LCX) coronary arteries and moderately stenosed, non-grafted right coronary artery (RCA). The patent left internal mammary artery (LIMA) to LAD graft with good insertion and the two occluded saphenous venous grafts (SVG) on the ascending aorta.
B Retrospective FAI Score and AI-Risk algorithm mapping of the RCA showing elevated perivascular inflammation. The dials are showing the 8-year predicted risk of a fatal cardiac event based on
  (i) the FAI Score alone, and
  (ii) an integrated score (AI-Risk algorithm) combining FAI, coronary plaque burden, and clinical risk factors. The dials are adjusted for age and sex and offer a visual summary of absolute and relative risk levels. The dials represent percentile-based risk levels: FAI Score is in the 87th percentile for age and sex, indicating elevated inflammation. The AI-Risk algorithm dial integrates this with clinical risk factors and coronary plaque data to yield an 8-year absolute risk estimate (18.4%). In these visualizations, percentile values indicate how the patient’s result compares to a reference population (e.g., 87th percentile = higher than 87% of matched individuals). The integrated risk combines inflammation (FAI Score), plaque burden, and clinical factors into a single predicted risk value.
C FAI Score relative risk, adjusted for age and gender

**Note**: The FAI Score and AI-Risk algorithm mapping shown are retrospective reconstructions performed post hoc using the 2014 CCTA data and were not available at the time of initial clinical decision-making

### Event and progression

Between 2014 and 2022, the patient remained clinically stable, with no further imaging investigations or significant anginal symptoms reported during follow-up. No revascularization procedures were pursued during this interval. In January 2022, the patient had been admitted to the hospital with non-ST segment elevation myocardial infarction (NSTEMI). Invasive coronary angiography showed an occluded RCA (non-grafted vessel, moderate stenosis in 2014), severe ostial left main disease, proximal/mid LAD occlusion, and OM branch occlusion. LIMA to LAD showed a normal calibre graft. Medical treatment was recommended. TTE at the time of the event showed regional wall motion abnormalities with a left ventricular ejection fraction (LVEF) of 40–45% with moderate MR. In February 2022, the patient was referred to a lipid specialist who prescribed the PCSK-9 inhibitor inclisiran as a further lipid-lowering strategy on top of ezetimibe. In November 2022, the patient presented with new heart failure symptoms including paroxysmal nocturnal dyspnea (PND) and peripheral edema. Bilateral moderate pleural effusion was present on high resolution chest CT. TTE confirmed the diagnosis of ischaemic cardiomyopathy with severely impaired left ventricular (LV) systolic function and severe ischaemic MR with raised end diastolic pressure. The patient initially responded to medical therapy. Potential interventions such as insertion of mitral clip or placement of an implantable cardioverter-defibrillator (ICD) were considered. However, the patient passed away shortly after his last hospitalization.

### Retrospective analysis

Retrospectively, FAI Score and AI-Risk algorithm analysis of the RCA were performed utilizing the CCTA from 2014 (Fig. 2B, C). FAI Score was 25.6 (87th percentile for age and gender), while the % 8-year risk for a fatal cardiac event based on the FAI Score along with clinical risk factors such as diabetes, smoking, hyperlipidemia, and hypertension, as well as the coronary atherosclerotic plaque burden as detected on CCTA was 18.4%, indicating a high residual inflammatory risk. Retrospective FAI Score and AI-Risk algorithm analysis were performed using CaRi-Heart^®^ Platform (Caristo Diagnostics Ltd., v2.4.2), based on validated methods previously described in the literature [1, 2].

## Discussion

We describe a case of a male patient with a history of CABG and a non-grafted RCA who, more than a decade later, presented with new onset angina. During the angina workup the non-grafted RCA showed only moderate stenosis on CCTA. However, 8 years later the patient suffers a NSTEMI with the invasive angiography showing an occluded RCA. FAI Score measurement and AI-Risk algorithm analysis, performed retrospectively, accurately predicted the increased risk for a future RCA event.

CCTA is currently used as the first-line imaging modality for stable chest pain according to clinical guidelines with the primary objective being the timely identification of individuals with obstructive CAD that need to undergo coronary revascularization [3, 4]. However, this approach identifies an abundance of patients without obstructive CAD for whom clinical management and relevant outcomes are not clear [5]. The early identification and appropriate management of individuals with inflamed coronary arteries, in the absence of obstructive CAD, is a long-standing unmet goal of preventive cardiology.

The use of the AI-Risk algorithm has been recently proposed in an ESC Clinical Consensus Statement as an addition to classic risk calculators, to provide a more comprehensive risk prediction when CCTA is available [6]. If available at the time, the use of the AI-Risk algorithm in the case presented here could have had important implications in the management of the patient. Specifically, had the elevated FAI Score and associated inflammatory risk been recognized in 2014, clinicians may have considered closer longitudinal follow-up, and earlier referral to a lipid specialist given the patient’s statin intolerance. While speculative, these interventions may have altered the progression of disease. Given the patient’s statin intolerance, alternative lipid-lowering agents beyond ezetimibe and inclisiran, such as bempedoic acid, could also have been considered as part of a personalized approach to reduce residual risk, though this option was not available until 2021. Furthermore, we acknowledge that the decision-making landscape in 2014 differed from today, and no direct changes in revascularization strategy would have been indicated based solely on FAI Score at that time.

Previous retrospective studies, such as the CRISP-CT and the subsequent development of the AI-Risk algorithm by Oikonomou et al. [2, 7], have demonstrated that perivascular FAI and its integration into a multi-parameter risk model can independently predict cardiovascular mortality in large patient cohorts. This case is intended to be hypothesis-generating and underscores the need for future prospective studies to assess the clinical utility and integration of the FAI Score and AI-Risk algorithm into routine cardiovascular risk stratification workflows. Broader validation of FAI Score and the AI-Risk algorithm in larger and more diverse populations is essential to confirm their predictive accuracy and clinical utility.

This is a retrospective single-patient case analysis, and the AI-Risk algorithm was applied post hoc using historical imaging. As the AI-Risk algorithm was originally derived from large population-based cohorts, its retrospective application to a single patient should be interpreted with caution, recognizing the inherent limitations in extrapolating population-level models to individual clinical decisions. The impact of scan variability, imaging protocol differences, and technical calibration factors, all of which may affect generalizability and risk prediction accuracy should be considered. Additionally, FAI Score and AI-Risk algorithm are not yet part of routine clinical workflows and were not available in 2014. This limits immediate clinical translation but highlights potential future applications in preventive cardiology.

In conclusion, this case report highlights the importance of measuring inflammation using the perivascular FAI Score in any coronary artery, regardless of the presence of obstructive CAD. While conventional CCTA is used to diagnose the anatomical severity of coronary stenosis, the FAI Score and AI-Risk algorithm provide prognostic information by quantifying vascular inflammation and long-term cardiovascular risk. These tools are not intended to replace traditional assessments but rather to complement them by identifying patients at high residual risk even in the absence of obstructive CAD. Despite their promise, these technologies are not yet fully integrated into routine clinical workflows, and their clinical adoption will require further validation through prospective, multicentre studies.

## Data Availability

The data supporting the findings of this case report are available in this published article. Further details are available from the corresponding author on reasonable request.

## Abbreviations

CABG: coronary artery bypass grafting
CAD: coronary artery disease
CCTA: coronary computed tomography angiography
D1: first diagonal branch
DSE: dobutamine stress echocardiography
FAI: fat attenuation index
ICD: implantable cardioverter-defibrillator
LAD: left anterior descending
LIMA: left internal mammary artery
LVEF: left ventricular ejection fraction
MR: mitral regurgitation
NSTEMI: non-ST segment elevation myocardial infarction
OM1: first obtuse marginal artery
PND: paroxysmal nocturnal dyspnea
PVAT: perivascular adipose tissue
RCA: right coronary artery
SVG: saphenous vein graft
TTE: transthoracic echocardiogram
